# A qualitative analysis of factors influencing the implementation of antiretroviral treatment adherence policy in Ghana: stakeholders perspective

**DOI:** 10.1186/s12961-023-01010-9

**Published:** 2023-06-14

**Authors:** Martha Ali Abdulai, Fraukje E. F. Mevissen, Veerle Marien, Robert A. C. Ruiter, Seth Owusu-Agyei, Kwaku Poku Asante, Arjan E. R. Bos

**Affiliations:** 1 Research and Development Division, Kintampo Health Research Centre, Ghana Health Service, P.O Box, 200, Kintampo-Bono East Region, Ghana; 2 Department of Work and Social Psychology, Maastricht University, P.O. Box 616, 6200 MD Maastricht, The Netherlands; 3 Department of Public Health, Municipal Public Health Service Rotterdam-Rijnmond, Rotterdam, The Netherlands; 4 Institute of Health Research, University of Health and Allied Sciences, PMB 31, Ho, Ghana; 5 Faculty of Psychology, Open University, PO Box 2960, 6401 DL Heerlen, The Netherlands

## Abstract

**Background:**

The Joint United Nations Programme on HIV/AIDS launched the 90-90-90 initiative. Failure to meet the target reflects the difficulties in successfully implementing HIV treatment policy. There are research gaps in exploring personal and external factors influencing HIV treatment in Ghana. To fill this gap, we explored individual and environmental (interpersonal, community and structural) factors influencing stakeholders' HIV treatment policy implementation in Ghana.

**Methods:**

Fifteen qualitative semi-structured in-depth interviews were conducted among representatives in different management positions at hospitals, health directorates, the Ghana AIDS Commission, the National AIDS and STI control program, and the National Association of People Living with HIV.

**Results:**

Using thematic analysis, the findings suggest that individual and environmental factors such as attitude towards policy, awareness of HIV treatment policy, training received on policy implementation, difficulties related to patient factors, alternate sources of HIV care, inefficient policy decision-making, monitoring and evaluation of HIV treatment policy, lack of HIV treatment policy implementation training, poor availability of logistics, policy and guidelines, infrastructure, organization of training, and staff availability may hinder successful HIV treatment policy implementation.

**Conclusion:**

Several individual and environmental (interpersonal, community and structural) factors seem to influence HIV treatment policy implementation. To ensure successful policy implementation stakeholders need to receive training on new policies, availability of sufficient supplies of material resources, inclusive decision-making, receive supportive monitoring of policy implementation, and oversight.

**Supplementary Information:**

The online version contains supplementary material available at 10.1186/s12961-023-01010-9.

## Background

In 2020, an estimated 37.6 million people worldwide were living with HIV, with approximately 27.4 million receiving antiretroviral therapy [1]. People living with HIV (PLWH) must take antiretroviral medications in a planned manner in order to achieve sufficient viral suppression [2]. In 2014, the Joint United Nations Program on HIV/AIDS launched the 90-90-90 initiative, with the goal of having 90% of all people living with HIV knowing their status, 90% of those diagnosed being on treatment, and 90% of those on treatment achieving viral suppression. The achievement of these goals by 2020 should accelerate the end of the HIV epidemic by 2030 [3].

In line with these global goals, Ghana adopted the treat-all-policy in 2016 by following the WHO Consolidated guidelines on the use of antiretroviral drugs for treating and preventing HIV infection (WHO 2016), which are implemented countrywide. This policy makes every PLWH eligible for treatment and thus increases the number of PLWH on antiretroviral therapy (ART) [4]. Following these targets, the Ghana AIDS Commission (GAC) estimates that in Ghana, 77% of the people diagnosed with HIV infection receive sustained antiretroviral therapy. The majority of them (68%) show viral suppression as a result of the treatment they received [5], which is, however, well below the target of 90% that show viral suppression by 2020. In this paper, we focus on the factors influencing the successful implementation of strategies to achieve 90% ART treatment adherence and viral load suppression.

In Ghana, HIV treatment policy stakeholders such as the Ghana AIDS Commission, National AIDS & STI Control Programme, and National Association of People living with HIV, Ghana Health Services (hereinafter referred to as stakeholders) are key agents in HIV treatment policy implementation and the promotion of ART adherence. They make policy decisions, oversee patient care and budgets, plan, direct and coordinate medical and health services. Stakeholders are important agents for leadership, supervision, performance feedback, monitoring the quality of care services, and the implementation of quality improvement processes [9, 7]. For example, the implementation of the HIV treatment policy in Ghana is led by the Ghana AIDS Commission (GAC), a supra ministerial and multisectoral body that is mandated among others to formulate HIV policies and strategies, mobilize, manage, and monitor resources, and foster linkages and networking of stakeholders [12, 13]. The National AIDS & STI Control Program (NACP) under the Ghana Health Service is the technical lead responsible for HIV treatment policy implementation. Together, the GAC and NACP work closely with non-governmental organizations (NGOs) such as the Ghana Network of people living with HIV (NAP +), community-based organizations (CBOs), regional, district/municipal directorates, and multinational and bilateral organizations to implement HIV policies, including HIV treatment [12, 13].

Effective policy implementation is critical for successful policy outcomes. Policy implementation is defined as a series of activities undertaken by program managers and others to realize the goals and objectives articulated in policy statements [14]. In this case, HIV treatment policy implementation. Stakeholders are incessantly looking for better ways to realize policy goals. Failure to implement policies can cause financial waste, political frustration, and disruption of health services for PLWH [16]. The underachievement of the UNAIDS 90-90-90 targets speaks to the challenges in the implementation of HIV treatment policy in Ghana.

**Fig. 1:**
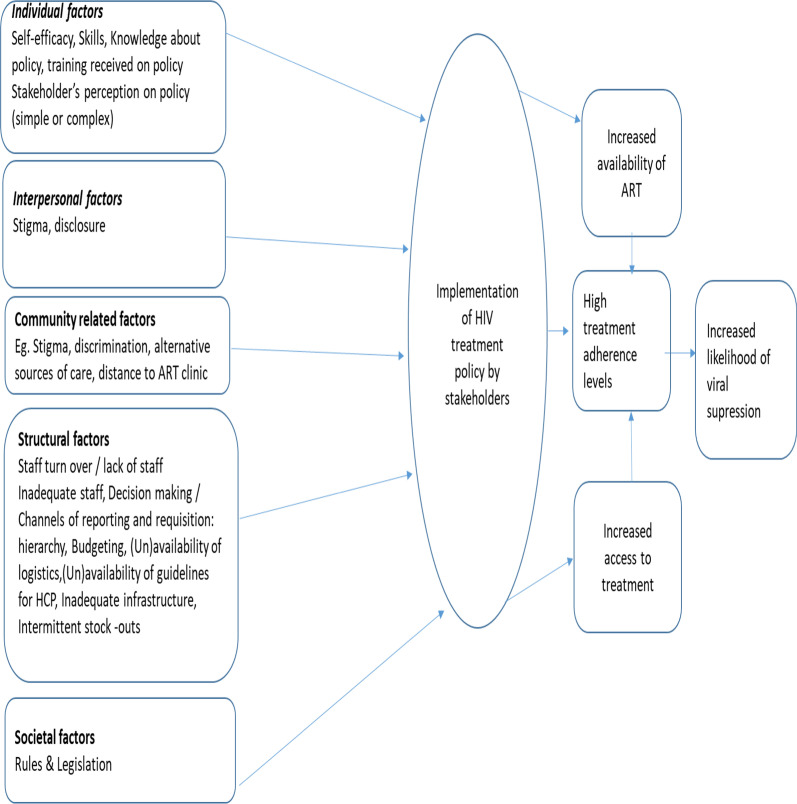
A logical framework of factors influencing stakeholders’ implementation of HIV treatment policy

For the promotion of successful policy implementation, it is essential to understand the factors that hinder and facilitate HIV treatment policy implementation by the various stakeholders. Using the socio-ecological model [17], we argue that the factors influencing stakeholders’ successful policy implementation are situated at the *individual, interpersonal, community, structural, and societal levels* [18, 19], as illustrated in Fig. 1(Appendix 1).

*Individual-level factors* such as knowledge, skills, and beliefs influence policy implementation. For example, stakeholders who are knowledgeable about the HIV treatment policy are confident in implementing the policy [21], while those with low knowledge may not have the requisite skills for the implementation of HIV treatment policy [22]. Stakeholders' understanding and clarity on policy implementation facilitates its implementation [24, 25]. Stakeholders who believe that HIV treatment policy is good because it aligns with their personal values are more likely to implement it than those who are not convinced [26].

Besides the individual factors, *interpersonal factors* also influence policy implementation. Undoubtedly, the behavior of the PLWH mediates the successful implementation of the HIV treatment policy towards adherence and viral suppression. For instance, stakeholders may not achieve HIV treatment policy outcomes of 90% treatment adherence and viral suppression due to a patient’s decision to discontinue their medication [27, 28]. Stakeholders are also influenced by the negative or positive norms of their colleagues in the implementation of HIV treatment policies. For example, when one stakeholder ignores monitoring of the viral load of a patient, colleagues may take a cue from it and do likewise, which over time becomes the norm [29]. These interpersonal factors may impede HIV treatment policy implementation and need to be addressed.

C*ommunity-level factors* including stigma, cultural norms, social support, and social networks influence stakeholders’ implementation of HIV treatment policies [30]. The NACP engages several people at the community level, including religious and traditional leaders, to get their buy-in for policies. When these leaders oppose or stigmatize a policy, it may be difficult to implement it within the community [30, 31]. Again, a community of people living with HIV who form a network may equally accept or oppose HIV treatment policies. Given that the network of PLWH is an important group for HIV policy implementation in Ghana, their opinion about a policy will influence its implementation by stakeholders [30, 31].

S*tructural factors* are also relevant to the implementation of HIV treatment policies. For successful policy implementation, it is important that stakeholders find quick and clever ways to overcome difficulties related to infrastructure, logistics, and personnel [32].

Finally, *societal factors* such as HIV-related punitive laws, policies, and practices, especially against key populations, impede stakeholders’ implementation of HIV treatment policies towards HIV treatment and viral suppression [33, 34]. Less progress has been reported towards HIV treatment and viral suppression in countries that criminalize key populations. These societal factors are, however, beyond the scope of this paper.

### The current study

Policy implementation is influenced by a myriad of personal and environmental factors that vary across developing and developed countries [35]. While available scientific publications on HIV policy in Ghana have so far focused on legal audits of HIV treatment [36], workplace HIV policy [37], and human rights of PLWH [38], there is a scarcity of research on the individual, interpersonal, community and structural elements that influence HIV treatment policy implementation in Ghana. To fill this gap, our study sought to explore the individual and environmental factors influencing stakeholders’ HIV treatment policy implementation using qualitative in-depth interviews with stakeholders that play a key role in the successful implementation of strategies to promote HIV treatment adherence. Knowing the perspectives of stakeholders will help in identifying targets for future interventions at the different ecological levels to promote successful implementation of HIV treatment policies and thus contribute to enhanced ART adherence and viral suppression among PLWH.

## Methods

This qualitative study used semi-structured interviews to get an in-depth understanding of the individual and environmental factors influencing the implementation of the HIV treatment policy among stakeholders.

### Study area and site selection

Ghana has 16 administrative regions and runs a decentralized system of governance from the national level to the district level. Similarly, the HIV treatment policy implementation follows this decentralized system, hence the need to engage stakeholders at all ecological levels. The study was conducted among stakeholders in the Bono East region and at the national level in Accra. The Bono East region is one of the sixteen regions in Ghana. The capital of the region is Techiman with, a population of 104,212. The Bono East Region covers a total land area of 39,557km2 with over 40 health facilities within the eleven municipal and district assemblies [40]. The region has an HIV prevalence of 1.78% [12]. The interviews in the region were conducted in three purposively selected hospitals (Kintampo South District, Kintampo North Municipal, and Techiman Holy Family Hospitals), district/municipal health directorates, and national offices of GAC, NACP and Ghana Network of people living with HIV (NAP +).

### Participants

**Fig. 2:**
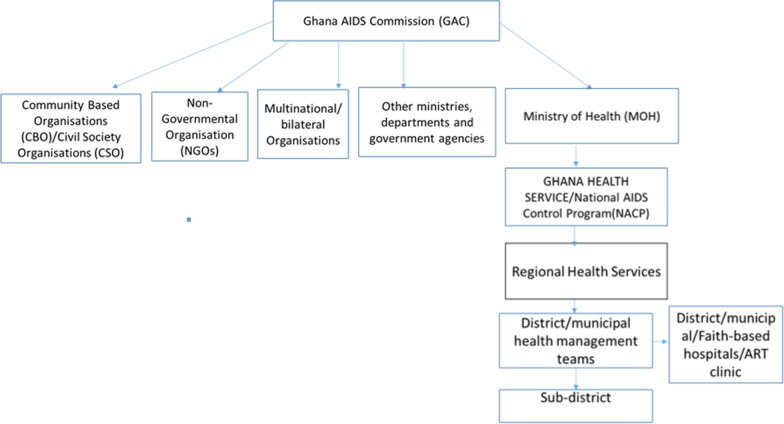
An organogram of stakeholders on HIV treatment policy implementation in Ghana. Source: National HIV/STI policy 2019

The study purposively sampled 15 stakeholders. Participants were eligible to participate in the study if they were 18 years and above, responsible for the coordination of health care activities including HIV/AIDS from the national, regional, district and sub-district level (see Fig. 2) and if they have no direct contact with PLWH in their role (see Table 1) for their specific roles in HIV treatment policy implementation. Their ages ranged between 31 and 57 years (median = 42, *M* = 45.7, *SD* = 9.7). The majority of the interviewees were males (*N* = 10) and five were female. The stakeholders worked in different management positions at hospitals, health directorates, and the National AIDS Control program (NACP), Ghana AIDS Commission (GAC), and the National Association of People Living with HIV (NAP +). Figure 2 (see Appendix 2) shows the hierarchy and different levels of the stakeholders in HIV care from which we selected our study participants.

**Table 1:** Program managers, number interviewed and their roles in policy implementation

Institution	Number of representative interviewed	Institutional Roles in HIV treatment policy implementation
District/municipal management Teams	6	Receive policy and guidelines from regional level, train healthcare providers and ensure policy implementation, overseeing, and monitoring HIV activities at the district level Coordinates HIV treatment, care and support at the district and facility levels
Hospitals Laboratory	6	Provides clinical care for HIV patients at the facility level, monitoring HIV viral load suppression through viral load testing, ordering logistics, ensures logistics availability for smooth implementation of policies, Generates hospital data for monitoring adherence, logistics management, stock-out of antiretroviral
Ghana AIDS Commission	1	Coordinates HIV treatment policy development. Formulate HIV/AIDS policy, direct and coordinate activities in response to HIV and AIDS Formulate policies and strategies on HIV and AIDS and determine program priorities * Provide high-level advocacy for HIV and AIDS prevention and control * Provide leadership in national planning, supervision, and support of the HIV and AIDS program * Plan and coordinate activities in relation to the national HIV and AIDS response * Mobilize, control and manage resources available for the achievement of the object of the Commission and monitor the allocation and utilization of the resources * Foster linkages among all stakeholders * Generate strategic information to influence policy, strategies, planning, and use of resources * Promote research and dissemination of information on HIV and AIDS and documentation of persons living with HIV and * Monitor and evaluate programs of the national HIV and AIDS response
National AIDS Control Program	1	Coordinates HIV treatment policy implementation at the regional and district levels overseeing, and monitoring HIV activities
Ghana Network of people living with HIV	1	Represents the voice of persons living with HIV Contribute to a broad range of activities from advocacy, human rights, ethics and legal considerations, and represent the key interface with the scientific community Convey expectations and concerns from the public for PLWH

### Data collection procedures

The approval to conduct the study was received from the heads of institutions. This was followed by a face-to-face meeting with each of them to explain the aim of the study (i.e., exploring the factors influencing HIV treatment policy implementation from the perspective of stakeholders). All eligible participants in managerial and decision-making positions who were approached by the researcher were willing to participate and were included in the study. Two participants were interviewed immediately upon recruitment. For the others, interviews were scheduled at an appropriate time and date. All fifteen interviews were carried out between April 2018 and June 2018 and took place in the offices of the participants. The first author is a female PhD student and an experienced qualitative researcher. The interviews were largely conducted by the third author who was graduate students from Maastricht University. Interviews were held in private spaces with, in some instances, brief interruptions by incoming people this however, only minimally affected the progress of the interview.

During recruitment and before each interview, the interviewer informed participants about the confidentiality, the voluntariness of their participation, and the possibility to refuse to answer any question or stop the interview without the need for explanation. Participants were told that, for privacy issues, their names are de-identified with unique identifiers. Participants were instructed to avoid mentioning their names or other names in the course of the interviews. Participants were asked for their consent to audio record the interview to make verbatim transcription possible, to which they all agreed. All the interviews were conducted in English. The interviews lasted between 40 and 60 min. We reached saturation by the 14th interview; one additional interview was carried out after which we ended the data collection since there were no new and distinct themes emerging.

### Interview protocol

The interview protocol was based on concepts derived from the empirical literature and theories on factors influencing the implementation of HIV treatment policies [27, 41, 42]. Interviewees were interviewed about their knowledge and beliefs about HIV, treatment policies and guidelines, attitude towards policy, adherence to policy, sources of policy, decision making structure and protection of PLWH rights (Additional file 1).

### Data coding and analysis

To analyse qualitative data, thematic data analysis was performed. The data analysis procedures followed Castleberry and Nolen's [43] thematic approach. The first author transcribed all fifteen audio recordings verbatim. The transcripts were reviewed for familiarity, accuracy, and completeness. The data were analysed using Atlas Ti software version 9. The first and second authors independently coded three interviews after which coding were compared. Any differences in coding were discussed until agreement was reached after which the first author coded the remaining interviews. The codes were then organized into possible themes. The authors addressed any disagreements in a series of meetings until reaching an agreement on the final themes. Resolving these disagreements resulted in consistency of coding and development of the final themes. A report was generated for each thematic category by the first author which was reviewed by the second author. The thematic categories were all summarised as HIV treatment policy implementation behavior of stakeholders. This is followed by the factors influencing this behavior at the individual, and environmental (interpersonal, community, and structural) levels.

## Results

Below we report on the determinants of HIV policy implementation in two broad categories: individual factors and environmental factors (including interpersonal, community, and structural factors). About implementation of the national HIV treatment policy participants said that the implementation of the policy/guideline is mandatory to implement “The policy is from above (national) and we have to follow it.” (HIV treatment policy stakeholder 2).

Even though health facilities can make minor changes in the implementation strategy to suit their particular needs.

“No, they [facilities] all apply the same one [policy/guidelines]. Other people only modify them a bit to suit their facility. You only change a few things. We also do that…” (HIV treatment policy stakeholder 6)

## Individual factors influencing HIV treatment policy implementation.

### Awareness of HIV treatment policy and guidelines

The majority referred to the national treatment guidelines or the 90–90-90 policy.

“You know, with the 90-90-90, we expect that 90% of the population should be tested and 90%, of those that test positive, should be set on treatment with 90% viral suppression.” (HIV treatment policy stakeholder 11)

The majority of stakeholders assumed high to very high levels of adherence among the PLWH at their location. If adherence percentages were given, they stated rates of 90% or above.

“Oh, adherence is good. I think it is 98%. I: 98%? R: Yes.” (HIV treatment policy stakeholder 2)

Participants mentioned that people who test HIV positive are linked to care to start antiretroviral therapy (ART). Once an individual starts treatment, s/he is supposed to be monitored for viral suppression every six months and subsequently annually.

“Yeah, we do the testing for them as they take the medication; we are supposed to check for their viral loads to see whether there is improvement while on the medication.” (HIV treatment policy stakeholder 10)

One participant said that the initial policy encouraged treatment support, i.e. someone PLWH trusted to disclose HIV status to help them take ART. However, in order to avoid delays, the current policy states that individuals who test positive are immediately put on ART [this may not give patients ample time to reflect and decide to stay on lifelong treatment for viral suppression]. This assertion may influence the implementation of this policy related to treatment adherence.

“….with the 90-90, once they are tested, they have to be enrolled in the ART…… Previously, they first get to be monitored; somebody will be monitoring their drug intake, but the donors are saying it is delaying the client.” (HIV treatment policy stakeholder 6)

### Attitude towards policy

The majority of the participants were satisfied with the HIV treatment policy guidelines because they considered them important, clear, simple, easy to follow and understandable.

“Ooh, it [guidelines] is simple, so easy to follow and understandable. Therefore, we are happy with that. It is not anything difficult, and anyone you take can do that.” (HIV treatment policy stakeholder 2)

They added that the current HIV treatment policy is useful and good, ensures uniformity of procedures across all health facilities, and improves care and adherence.

“Yeah, it [guidelines] improves care, because most of them will hide and then come at the terminal stage of the disease. The earlier you start, the better.” (HIV treatment policy stakeholder 3)

Nonetheless, some participants stated that, while the 90-90-90 UNAIDS goal eliminates delays previously caused by laboratory tests, the current policy is worsening adherence because PLWH default on appointments with excuses [because they no longer have to go through rigorous adherence counseling before starting treatment].

“The issue is that now they have introduced the 90-90 policy, it is worsening the situation. The day they are tested; they should be enrolled and start ART; but some default on the appointment time; example If they were supposed to come on the 15th of last month you see the clients coming at the end of this month; with a lot of excuses.” (HIV treatment policy stakeholder 6)

Some participants were sceptical about the 90–90-90 policy. While one stated that potential patients are immediately put on drugs with the current policy before the CD4 count confirmation,

“……with the 90-90, once they are tested, they have to be enrolled to the ART.” (HIV treatment policy stakeholder 5)

Another bemoaned that PLWH are challenged with logistics. If indeed the government is committed to the achievement of the 90-90-90 policy, they should be committed to getting all the logistics available to ensure a smooth transition from the first, second and third 90s.

“The 90-90-90 agenda is to ensure that 90 percent and those of us who are already in the health system; we do not have sufficient logistics to cover even the fewer of us that are already on treatment. Now we are talking about 90 percent coming out to know their HIV status. If truly the Ghana government signed the MOU with the United Nation to ensure that the 90 percent persons living with HIV in Ghana know their HIV status. Then whatever it takes this 90 percent to know their status the government should also be committed to it. It would not be the case that oh we are struggling to get all these 90 percent and when they get to treatment then it becomes a barrier for them to access treatment; I do not think this will help. The second 90 said all those 90 percent when they know their status they should be put on treatment. It means that treatment includes monitoring the viral load system, the CD4 count, the liver function and the other entire test that associated with the treatment…” (HIV treatment policy stakeholder 14)

### Policy-related training received by stakeholders

The majority of the participants said they received training whenever there were new policies or guidelines related to antiretroviral therapy and logistics management, among others.

“Yes. Because normally when new policies are coming, we are called. They train us and we come back and train the other workers.” (HIV treatment policy stakeholder 13)

Some of the participants called for more new and refresher training for new and old staff respectively, due to the high staff-turnover. They went on to say that due to the unique nature of HIV therapy, there is a need to put counselors through systematic training and train all professionals on how to manage HIV.

“What I know is that because HIV is a program, not all clinicians have been trained on how to manage HIV, so yes [more training is needed for all physicians].” (HIV treatment policy stakeholder 10)

## Environmental factors influencing HIV treatment policy Implementation

### Stakeholders’ difficulty in implementing policy due to patient-related factors

Some stakeholders indicated that patients' non-attendance of clinic appointments, thus default adherence to medication, affects treatment adherence and viral suppression. Some of the reasons they ascribed to patients defaulting include non-disclosure, stigma, and distance to ART/financial challenges [due to these challenges, patients do not show up to receive medication, which influences the achievement of treatment adherence and viral suppression]. Some participants explained the long travel distance because of the communities’ being far away from the clinics:

“Money for them to pay the T&T to the facility is a challenge, we have long distances between our facilities. PLWH cannot walk, and the money is not there, they will stay home and default.” (HIV treatment policy stakeholder 12)

Many participants said stigma is a key issue-influencing treatment adherence for PLWH and subsequently fraught stakeholders’ policy implementation, [When PLWH are stigmatized, they may not adhere to their medication, hence high viral load], because of stigma PLWH tend to hide their HIV status, hide medications and travel far distances for medication.

“I will say the major challenge is stigma and a key issue. So most people do not open up about HIV because of the stigma ……. most people do not take the medications in the town they live in. For example, if the patient lives in Techiman and takes their medication at Techiman Holy Family Hospital people may come to know their HIV status, they would rather go to faraway places like Sunyani…..where nobody knows them.” (HIV treatment policy stakeholder 10).

### Alternative sources of care

The majority of stakeholders expressed concern about the difficulty of implementing HIV treatment policies due to alternative sources of care such as local medication and faith healers. They explained that PLWH are turning to these alternative sources in the hope of finding a cure and in order to alleviate the difficulties associated with taking antiretroviral medication for the rest of their lives. Unfortunately, this causes such PLWH to retrogress [viral suppression] and, in some cases, to die.

“Some would come and tell you, ‘’oo, my pastor says he has prayed for me, and for that matter, the sickness is no more there.’’ This person will be at home for some years, and comes back terminally ill, at which point we can only do little, most of them, even die. In 2015-2016, HIV/AIDS was the highest cause of mortality in this hospital.” (HIV treatment policy stakeholder 9)

Several participants emphasized that superstitious beliefs about HIV, faith healers, especially pastors in the media space, who claim to cure HIV hinder HIV treatment policy implementation. They bemoaned that even though they educate the populace on HIV treatment, faith healers cause confusion among people by contradicting this information.

“Yes, we do education all right but this spirituality is now becoming an issue in Ghana. Moreover, because of the mass media, people have access to radio stations and can spread all sorts of information. Somebody said untreated candidiasis will turn into syphilis, if untreated which will eventually turn into HIV. The way these people speak with passion, the HIV patients tend to become confused as to which one to believe.” (HIV treatment policy stakeholder 10)

### Availability of staff

The majority of stakeholders said there was a lack of or inadequate staff at all levels to provide HIV [treatment] services, especially counsellors and clinicians. This staff shortage or inadequacy overburdens available personnel, causing them to overlook the demands of HIV treatment policy implementation.

“…..we are supposed to run the laboratory 24 hours. We need to run night shifts, and all those things. Here is the case; we are not able to do that because of the problem of staff strength…” (HIV treatment policy stakeholder 16)

### Policy decision-making

Most participants stated that decision-making and the channels of reporting on HIV treatment policy are hierarchical from the national, to the region, to the districts/municipalities and then to the sub-districts. The districts/municipalities, are mostly the policy-implementing sites of the decisions that are made at the national level. When reporting, stakeholders report to the regional, who also report to the national.

“We also have our bosses from the national level, regional level and the districts. Most often, we at the district level implement the decisions that come from above, so when it comes to us locally, then we will now look at the national policy. If it is suitable for us, then we go by it, but because of our setting and some peculiar challenges, we make certain amendments to suit our situation.” (HIV treatment policy stakeholder 5)

At the facility level, administrators and medical superintendents, in consultation with management, make major decisions about HIV treatment policy implementation. District/municipal directors sometimes endorse these decisions before they are implemented.

“The district director is the general overseer. He approves of whatever decision we want to carry out. Even though I am a coordinator, if I want to do anything, he has to endorse it before I can carry it out, I cannot do things on my own.” (HIV treatment policy stakeholder 12)

One participant said that national-level decisions about policy implementation could not be challenged; you can only give feedback at the district, regional and sometimes national level. If other facilities report, same results, further research may be launched by the GHS through its research centres.

“We are policy implementers. At Ghana Health Service, we implement policies from the Ministry of Health, so when the policy is in and you are trained, you have to implement it and then give your feedback to them. You evaluate and then give feedback. When it is coming, you cannot object.” (HIV treatment policy stakeholder 11)

### Monitoring and supervision of HIV treatment policy

The majority of participants monitored and evaluated the implementation of the policies based on written reports. They mostly focused on “the numbers” (how many people came for a test, do we have sufficient medication, etc.). Only one person very clearly stated that she visits the facilities to see how people are doing their work and if patients are satisfied with the care they receive.

“Once a while I do visit that place. I do interview clients about their charges and their services: are they satisfied with the services that they are providing… Even this morning I went to the maternity unit, the children’s ward, the lab and the dispensary, we came back through ART corner, the ANC, and OPD.” (HIV treatment policy stakeholder 8)

One participant said that the Ghana AIDS Commission in collaborations has carried out joint implementation support-monitoring visits to know what is happening at the field level. Findings from the field are then discussed in this quarterly review meetings and feedback to the implementing partners. However, they have not effectively monitored these policies.

“We have not effectively also monitored these policies.” (HIV treatment policy stakeholder 13)

### Organization of training on HIV treatment policy

The majority of stakeholders reported that policy-related training is often organized at the national, regional, and district/municipal levels and participants are expected to train people in their facilities upon return. They also stated that the training is primarily designed for new staff to become acquainted with HIV-related issues, including treatment, and that refresher training is designed for existing staff to learn about new policies and guidelines for practice.

“The refreshing training for the old ones and then the training for the new ones who are coming in the system.” (HIV treatment policy stakeholder 12)

### Availability of logistics

Some stakeholders stated that logistics such as antiretroviral medicines were mostly available for successful policy implementation. Most others did, often at first, stressing that it was all okay and “people are doing their best”. Nevertheless, in the end, almost all participants did mention shortages of logistics such as patient folders, medication, and reagents as well as finances and transportation options (vehicles, motorbikes). Sometimes it was a bit unclear whether there was or was not a shortage because they contradicted themselves throughout the interview, or they stressed the shortage was in the past but no longer.

“I think the national AIDS control program and the Ghana AIDS Commission are doing their best, we have up-to-date drugs and test-kits. We have folders as well. At times, the folders run out of stock….Moreover, the hospital management is doing their best photocopying of some of the sheets so for those who will come as newcomers when we do not have folders from the NACP… At times, we have challenges with the vehicle reaching out to people at home.” (HIV treatment policy stakeholder 9)

In the event of a drug shortage, all patients receive a one-month supply rather than a three- or six-month supply, implying that patients must travel much more frequently to obtain a new supply.

"For instance, suppose you arrive and HCP informs you that "oooh, there is stock out, so I have to distribute the few available containers with everyone visiting the facility today." ……This also leads to many defaults because when a person has to pay their transportation to the ART center, receives only two weeks' medications, not everyone can afford returning to take more. Some people could give up and say, "Why should I?" and if I die, I die. We have seen a lot of them default due to transportation cost.” (HIV treatment policy stakeholder 14)

One participant stated that the major challenge with the erratic supply was the fact that the antiretroviral is not readily available in the open market and has to be supplied by the national program.

“I also talked about the inadequate and erratic supply of drugs because sometimes they are unavailable at the national level...and you cannot buy them from outside because they are not sold at the open market….” (HIV treatment policy stakeholder 12)

The majority of the participants bemoaned the lack of PCR for patient viral load monitoring [this affects the implementation of the 90% viral suppression goal]. The PCR was unavailable and samples within the region had to be sent to a central location from all health facilities in the region and beyond for testing. They added that the few machines and equipment available periodically break down or are faulty, which delays patient test results. “Yes [it will take at least one month to get the results back].” (Stakeholder 16).

### Availability of policy and guidelines

Some of the participants stated the availability of several guidelines, including those for adult and paediatric antiretroviral therapy. These protocols and guidelines were said to be in the ART centres. One participant indicated that even though the Ghana Health Service provided general guidelines, sometimes it was difficult to follow because they did not have the gadgets to follow through. As such, they choose and pick based on what equipment are available.

### Infrastructure

Few participants said the space available for storing enough ART is inadequate, which distracts care, hence ensuring treatment adherence. They bemoaned that it is even more so if you have to attend to a patient with HIV/TB coinfection.

“The clinic is too small and not well ventilated. Uhm…, the records room is not spacious. Moreover, you know, most of them [PLWH] have TB or co-infections. I worry about the health of others given the small nature of cubicles with improper ventilation.” (HIV treatment policy stakeholder 9)

## Discussion

Ghana has made some strides towards the implementation of HIV treatment policy though the targeted 90-90-90 was underachieved [5]. The present study provides insights into individual and environmental factors (interpersonal, community and structural) that may influence the implementation of the HIV treatment policy among policy stakeholders using in-depth interviews. The findings suggest that individual factors, including attitude towards policy, low awareness of policy, training received on policy implementation, and environmental factors such as difficulty due to patient-related factors, alternate sources of HIV care, availability of staff, organization of clinic, decision-making, monitoring and evaluation of policy, and organization of training on HIV treatment policy, may influence HIV treatment policy implementation.

Among these factors, training received on HIV treatment policy implementation, decision-making, availability of logistics, monitoring, and supervision seem to have the strongest influence.

Findings from our study indicate that HCPs receive both pre- and in-service training on HIV treatment policies when new policies are introduced. This training is essential for HCPs’ knowledge and skills for HIV treatment policy implementation towards medication adherence and viral suppression. This importance of pre- and in-service training is similar to studies in West Africa and in Asian study investigating stigmatizing attitudes towards people living with HIV/AIDS by doctors and nurses in Vientiane in Lao PDR [44, 45]. In contrast, Rowe and colleagues note that an increase in training alone does not automatically mean HCPs will implement the HIV treatment policy [46]. We argue that, indeed pre-service training alone may not be enough for HCPs to implement HIV treatment policies given the changes that occur in the field. This training may not always be adequate for HCP to function in their roles and may not be responsive to the current technology in HIV treatment [6, 7, 45]. As such, it is important to continuously train HCPs on HIV treatment policies to be abreast of changing trends and technology in the field. Scoping reviews in LMIC identified in-service and post training support as essential for developing the capacity of HCPs for continuous performance. It could be useful for future research to assess the role of training and content of training in policy implementation as a component of an overall monitoring and evaluation of HIV treatment policy in Ghana.

Our results show that even though participants mentioned that they monitor HIV policy implementation, they mostly did so through reports they received from the field and hardly ever visited to access the fidelity of the reports they received. We contend that HCPs may not implement HIV treatment policies the way they should and therefore require continuous monitoring and evaluation to track and identify gaps that require improvement in the 90-90-90 policy for successful ART adherence and viral suppression among PLWH [47]. The lack of or limited monitoring and evaluation for HIV treatment policy may result in voluntary implementation of the policy as identified by Phulkerd and colleagues [7]. Similarly, the end term evaluation of the strategic information component of the NSP 2011–2015 noted weaknesses in monitoring and evaluation of HIV and AIDS at the sub-national levels and most of the evaluations are undertaken at the national level [8]. Although participants indicated some level of monitoring and evaluation, there is a need for more facilitative supportive visits to monitor the fidelity of the HIV treatment policy in all health facilities.

The availability of ART at the time of the study was a huge incentive for HIV policy implementation towards ART adherence and viral suppression. Similar to this finding the London “keep doing it” campaign that ensured universal HIV treatment free of cost to patient by UK’s National Heal Service among other factors helped in treatment adherence and viral suppression [15]. This notwithstanding, of note were periodic shortages of drugs, reagents for viral load testing, and transportation challenges which could stall the implementation of the HIV treatment policy. These inadequate or periodic shortages of drugs and supplies, equipment, transport, and infrastructure undermine the health system’s ability to support the successful implementation of the HIV treatment policy. Our finding is corroborated by Dalinjong and colleagues in Ghana who synthesised facilitators and barriers with the implementation of the free maternal health policy [20]. Again, a national policy review in six sub-Saharan African countries noted that despite high levels of HIV treatment policy implementation, facilities reported ART stock-outs in the past three months [23]. Erratic supply of ART comes with negative consequences on treatment outcomes and patients’ motivations to remain on HIV treatment. Indeed, a strong health system is requisite for successful HIV policy implementation as it provides the workforce, equipment, drugs and supplies, transport, information, responsive services, monitoring and supervision [39]. The successful implementation of the HIV treatment policy requires a continuous supply of antiretroviral and reagents among other logistics for PLWH to maintain medication adherence and viral suppression.

Our study findings point to the fact that decision-making and the channels of reporting are hierarchical from the national level, to the regions, to the districts/municipalities, and then to the sub-districts. Reporting is from the sub-district up to the national level. The districts/municipalities are mostly the implementing sites of the decisions that are made at the national level. Even though this top-down approach comes with some advantages, when people are involved in decision-making about the implementation of the HIV treatment policy, they feel greater control and own the outcomes of such decisions [48]. Some participants' observance of hierarchy in HIV treatment decision-making that seems not to involve them may influence their behavior towards the HIV treatment policy implementation towards the 90–90-90 goal as corroborated by Ogunlayi and Britton [48] and requires the needed attention.

The findings of the study have some potential limitations. First, the findings of this study are not fully generalizable given the use of qualitative interviews and the number of stakeholders interviewed. Nonetheless, the use of in-depth interviews allowed for a detailed examination of individual and environmental factors that seem to influence HIV treatment policy implementation. To the best of our knowledge, this is one of the first studies in Ghana to investigate these factors in the implementation of HIV treatment policies. Future studies should use systematic approaches such as intervention mapping to further explore the fidelity of HIV treatment policy implementation from ART clinics.

## Conclusion

Several individual, interpersonal, community, and structural factors seem to influence HIV treatment policy implementation. These include training, monitoring and supervision, the availability of material resources, and decision-making. These factors influencing HIV treatment policy implementation could serve as targets for initiatives to stimulate stakeholders’ commitment to successful HIV treatment policy implementation. We suggest that a systematic approach such as intervention mapping be used to develop, implement, and evaluate HIV treatment policy implementation programs.

### Supplementary Information

**Additional file 1.**

## Data Availability

The data underlying this article will be shared on reasonable request to the corresponding author.

## Appendix 1

See Fig. 1.

## Appendix 2

See Fig. 2.

## Abbreviations

AIDS: Acquired immunodeficiency syndrome
ART: Antiretroviral therapy
CBOs: Community-based organizations
HIV: Human immunodeficiency viruses
GAC: Ghana AIDS Commission
NACP: National AIDS & STI Control Program
NAP +: Ghana Network of peoples living with HIV
NGOs: Non-governmental organizations
PLWH: People living with HIV
