# Supplementary material for: A qualitative analysis of factors influencing the implementation of antiretroviral treatment adherence policy in Ghana: stakeholders perspective
Source: Health Res Policy Syst. 2023 Jun 14;21:54. doi: 10.1186/s12961-023-01010-9 (PMC10265905; doi:10.1186/s12961-023-01010-9)
Supplement: Supplementary file 1 — Additional file 1. [file 12961_2023_1010_MOESM1_ESM.doc]

**Appendix 3 : Indepth Interview (IDI) guides**

**AUTHORS INSTITUTE**

**Determinants of antiretroviral treatment adherence and psychological wellbeing of people living with HIV: A case of antiretroviral centres in the Brong Ahafo region of Ghana**

**Interview guide for District and hospital management**

**Date of interview:** ­­­____ /____/____ (DD /MM/ YYYY)

**Start time:** **...........................** **End time:** **………………………………….**

**District:** _________________ **ART CLINIC:** …**………………………**

**Number of years worked.....................Number of years worked in HIV................................**

**IDI type of person:** **…………………….....................................................................**

**IDI Number (Circle): 1/2/3/4/5**

**Participant Age:…………… Levels of education**: **………………………………..**

**Gender:………**

**Introduction**

My name is …... from (authors institute). We are doing a study on ART treatment adherence among PLWH in the Brong Ahafo Region of Ghana. As a [manager/director/…] of health services in the district, your opinion and vision is very important. We would like to hear from people like you how PLWHIV deal with HIV and taking their ART medication. We also like to hear your experience with healthcare delivery for PLWHIV who visit the hospital and the ART centres. During this interview, I will ask you different questions, these questions are about your opinion and experience, and there are no right or wrong answers. If a question makes you feel uncomfortable, you do not have to answer it. You may stop or interrupt the interview at any time without having to explain yourself.

I would like to record the interview because this is helpful for the analysis later on. Your name will not be used/recorded during the interview and will never be included in any transcripts or reports, neither in the analysis or in the research paper, and I will ensure that third parties will not be able to identify you. Is it okay for you if I record the interview?

This interview will take approximately 1 hour. If anything is unclear or you need a short break, please let me know. Before we start, I would like to ask you to read this form of consent and sign it please. Do you have any questions so far?

***Introductory questions:***

Can you tell me a bit about yourself? (Probe current and previous jobs)

- What is your age?
- Gender
- Do you live in this community?
- Are you religious? / What religion?
- What is your educational background and previous work experiences?

**Management of ART clinic**

Can you tell me your experience with managing this ART clinic?

- What is your current position at the ART clinic
- How long have you been the director/manager?
- Can you tell me a bit about your role as health care manager of the ART clinic?
  - Do you enjoy your work?
  - What do you enjoy most? Why?
  - What do you dislike? Why? How do you deal with that?
- What is going well?
- What are difficulties you encounter?
  - Is there anything you can (and want to) do about it?
- For whom are you responsible (who do you manage) and who is responsible for you (who is managing you).

**PLWHIV Experiences & ART adherence**

- Can you tell me your experiences with working with PLWHIV?
- What is your level of involvement?
- What in your opinion is the biggest struggle PLWHIV have to deal with?
- Why?
- What could be possible solutions?
- Who is responsible for these solutions?
- What would you say is the ART centre’s contribution to ART adherence
- Can you tell me how the ART centres contributes to the positive experiences of PLWHIV
- Why that? Any other roles?
- Do you feel the centre is currently succeeding in that role?
  - If yes: how/examples?
  - If not: why not? What is needed? Whose responsibility is that? What is needed to make that happen?
- Do you have any idea about the level of adherence among your patients?
  - What do you think are the main problems for PLWHIV to adhere to medication?
- What in your opinion are the Solutions?
- Does the ART clinic play a role/has responsibility in ART adherence?
  - If yes: what & why
  - If not: why not? Who has responsibility?
  - What is needed to increase adherence levels? Who should organize that? Do you think you can play a role in that?
- Do you personally feel responsible for the ART adherence levels? (Why (not)?)
- Can you tell me about how HIV services and health care delivery is funded in this district/ hospital/ ART clinic?
  - What would be a reasonable amount of money to really make a difference?

**Health facility work environment & structure**

Can you tell me how the care for PLWHIV is organized in the district/hospital (Probe ART clinic?)

- What’s good/ going well about the HIV care (Probe ART clinic)
- What needs improvement regarding the HIV care (Probe ART clinic)
- Can you describe the facilities available/unavailable for providing PLWHIV health care?
  - (Probe Gloves, PrEP)
  - Can you tell me about the supply and availability of protective supplies for providing care?
- How supportive is the environment for the Health care workers who provide care to PLWHIV at this facility?
  - Influence policy/community
  - Facility-related factors
  - Patient-related factors
  - Education/training
  - Role of management/your role
- Can you describe the decision-making structures in providing HIV/AIDS care in the ART clinic?
- What do you think of this decision making structure?
- How does it affect service delivery?
- How do you deal with it?
- Can you tell me about any collaborations towards HIV care and treatment between this ART clinic and other stakeholders in the community?
  - Other ART clinics?
  - district/hospital?
  - (family, community leaders, psychosocial centre, religious leaders and local government towards PLWHIV)
- What do you think would be a good approach to achieve successful service provision for PLWHIV?
- Why?
- Examples?

**Policies & Guidelines**

- Are there any policies/guidelines that guide the work/care/treatment provided at the ART clinic?
- Can you describe them?
- What do you think of these policies?
- Are they necessary and useful?
- If yes: examples
- if not: why not? What is needed?
- Where are the policies based on?
- Who developed them / what are the sources?
- For whom are these guidelines?
- Do they adhere to these guidelines?
  - Why?/ why not? (Satisfied/ appreciation?)
  - Does anybody check if employees follow the policy/guidelines?
- Are the policies for HIV care and treatment based on any regional or national guidelines?
- What are they?
- Would you say these policies and guidelines protects the rights of PLWHIV (If Yes/No Why?)
- Are PLWHIV given equal treatment as other patients

Would you like to add anything?

**End interview and thank participant/s**

**AUTHORS INSTITUTE**

**Determinants of antiretroviral treatment adherence and psychological wellbeing of people living with HIV: A case of antiretroviral centres in the Brong Ahafo region of Ghana**

**Interview guide for National Policymaker’s interview guide**

**Date of interview:** ­­­____ /____/____ (DD /MM/ YYYY)

**Start time:** **...........................** **End time:** **………………………………….**

**District:** _________________ **ART CLINIC:** ………..**…………………**

**Number of years worked.....................Number of years worked in HIV................................**

**IDI type of person:** **…………………….....................................................................**

**IDI Number (Circle): 1/2/3/4/5**

**Participant Age:…………… Levels of education**: **………………………………..**

**Gender:………**

**Introduction**

My name is …... from (authors institute). We are doing a study on ART treatment adherence among PLWH in the Brong Ahafo Region of Ghana. As a [policy maker/policy stakeholder…] on HIV prevention, treatment, care and support in Ghana, your opinion and vision is very important. We would like to hear from people like you how PLWHIV deal with HIV and taking their ART medication. We also like to hear your experience with policy making, implementation and healthcare delivery for PLWHIV who visit the hospital and the ART centres. During this interview, I will ask you different questions, these questions are about your opinion and experience, and there are no right or wrong answers. If a question makes you feel uncomfortable, you do not have to answer it. You may stop or interrupt the interview at any time without having to explain yourself.

I would like to record the interview because this is helpful for the analysis later on. Your name will not be used/recorded during the interview and will never be included in any transcripts or reports, in the analysis nor in the research paper. I will ensure that third parties will not be able to identify you. Is it okay for you if I record the interview?

This interview will take approximately 1 hour. If anything is unclear or you need a short break, please let me know. Before we start, I would like to ask you to read this form of consent and sign it please. Do you have any questions so far?

***Introductory questions:***

Can you tell me a bit about yourself? (Probe current and previous jobs)

- What is your age?
- Gender
- Do you live in this community?
- Are you religious? / What religion?
- What is your educational background and previous work experiences?
- Current position at Ghana AIDS Commission (GAC)
- How long have you been in this position at GAC

**History and Origin of HIV Policies & Guidelines**

Can you tell me about the history and origin of the HIV response/policies/guidelines in Ghana

What has been some of the successes chalked over the years in the HIV response? Probe

- What do you think of the response/policies/guidelines
- Are they necessary and useful?
- If yes: examples
- if not: why not? What is needed?
- Where are the policies based on?
- Who developed them / what are the sources?
- For whom are these guidelines?
- Would you say the implementers adhere to these guidelines?
  - Why?/ why not? (Satisfied/ appreciation?)
- Do you engage in Monitoring and Evaluation of the policy guidelines you develop for the HIV response? How often and at what levels do you engage the implementers (Probe: regional, district, sub-district, facility-level)
- Can you tell me the sources of HIV care and treatment policy (probe: any regional or national guidelines?
- If yes what are they?
- Would you say these policies and guidelines protects the rights of PLWHIV (If Yes/No Why?)
- Are PLWHIV given equal treatment as other patients in receiving care
- What is the role of GAC in the HIV/AIDS response in Ghana
- What are the implications of the current policy for HIV prevention, treatment and care
- Can you tell me how GAC is involved in policy implementation

Can you tell me some of the challenges/facilitators of the National HIV RESPONSE?

Probe:

- What strategies have been put in place for 90 90 90 and the SDG’s,
- What are the targets for 2018
- What are the sources of funding for HIV activities in Ghana,
- Is there a dedicated fund for tackling the HIV response in Ghana

**Collaborating with other institutions**

- Can you share some experiences in collaborating with other institutions (Probe for past and present)
- What would you say have been the outcomes of these collaborations

**PLWHIV Experiences & ART adherence**

- Can you tell me your experiences with working with PLWHIV at the National level?
- What is your level of involvement?
- What in your opinion is the biggest struggle PLWHIV have to deal with?
- Why?
- What could be possible solutions?
- Who is responsible for these solutions?
- What would you say is the ART centre’s contribution to ART adherence
- Can you tell me how the ART centres contributes to the positive experiences of PLWHIV
- Why that? Any other roles?
- Do you feel the ART centres are currently succeeding in that role of ensuring ART initiation, adherence and viral supression?
  - If yes: how/examples?
  - If not: why not? What is needed? Whose responsibility is that? What is needed to make that happen?
- Do you have any idea about the level of adherence among your PLWH
  - What do you think are the main problems for PLWHIV to adhere to medication?
- What in your opinion are the Solutions?
- Does the ART clinic play a role/has responsibility in ART adherence?
  - If yes: what & why
  - If not: why not? Who has responsibility?
  - What is needed to increase adherence levels? Who should organize that? Do you think you can play a role in that?
- Do you personally feel responsible for the ART adherence levels? (Why (not)?)
- Can you tell me about how HIV services and health care delivery is funded in the district/ hospital/ ART clinic?
  - What would be a reasonable amount of money to really make a difference?

**Health facility work environment & structure**

Can you tell me how the care for PLWHIV is organized in the district/hospital (Probe ART clinic?)

- What’s good/ going well about the HIV care (Probe ART clinic)
- What needs improvement regarding the HIV care (Probe ART clinic)
- Can you describe the facilities available/unavailable for providing PLWHIV health care?
  - (Probe Gloves, PrEP)
  - Can you tell me about the supply and availability of protective supplies for providing care?
- How supportive is the environment for the Health care workers who provide care to PLWHIV at this facility?
  - Influence policy/community
  - Facility-related factors
  - Patient-related factors
  - Education/training
  - Role of management/your role
- Can you describe the decision-making structures in providing HIV/AIDS care in the ART clinic? Probe
- Can you share your thoughts on this decision making structure?
- How does it affect service delivery?
- How do you deal with it?
- Can you tell me about any collaborations towards HIV care and treatment between this ART clinic and other stakeholders in the community, other departments and ministries ?
  - Other ART clinics?
  - District/hospital?
  - (family, community leaders, psychosocial centre, religious leaders and local government towards PLWHIV)
- What do you think would be a good approach to achieve successful service provision for PLWHIV?
- Why?
- Examples?

Would you like to add anything?

**End of interview and thank participant/s**
